# Supplementary material for: Single-cell RNA sequencing reveals a heterogeneous response to Glucocorticoids in breast cancer cells
Source: Commun Biol. 2020 Mar 13;3:126. doi: 10.1038/s42003-020-0837-0 (PMC7070043; doi:10.1038/s42003-020-0837-0)
Supplement: Supplementary file 1 — Supplementary Information [file 42003_2020_837_MOESM1_ESM.pdf]

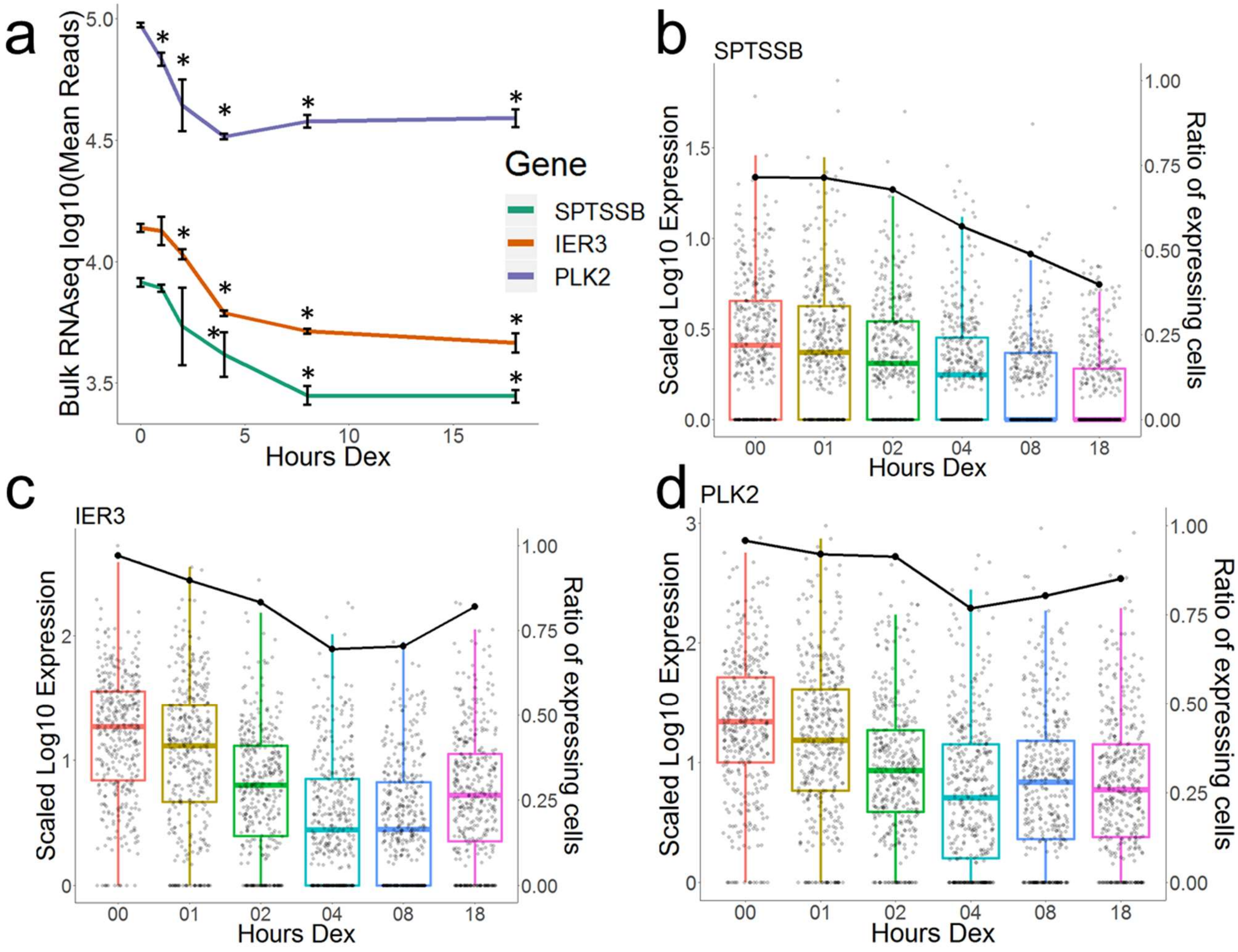

Supplemental Figure 1: a) Expression patterns for SPTSSB, IER3, and PLK2 across the Dex time course in the bulk RNAseq. Asterisks = p-value < 0.05. B-D) Scaled Log10 expression (box and scatter plots) and ratio of expressing cells (black dot/line plot) for SPTSSB, IER3, and PLK2 in scRNAseq. Each dot in the scatter plot represents an individual cell.

a

Bulk RNAseq DEGs

	Dex1hr	Dex2hr	Dex4hr	Dex8hr	Dex18hr
# DEGs	172	735	1370	2056	2417
# up	141	586	932	1223	1394
# down	31	149	438	833	1023

b

scRNAseq DEGs

	Dex1hr	Dex2hr	Dex4hr	Dex8hr	Dex18hr
# DEGs	77	94	167	275	285
# up	36	58	127	197	210
# down	41	36	40	78	75

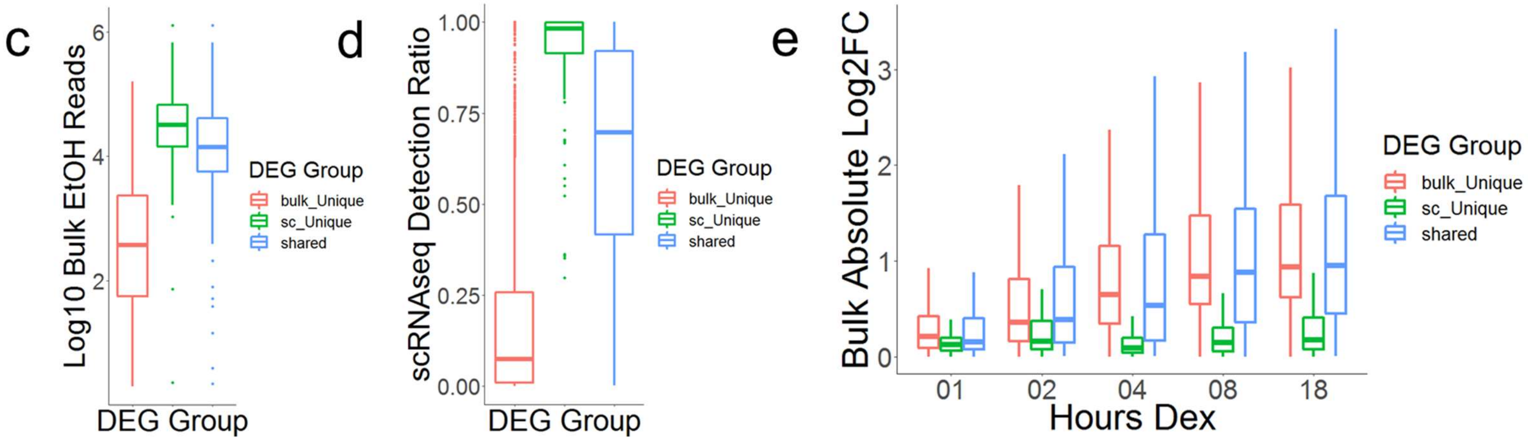

Supplemental Figure 2: a-b) Tables showing the number of DEGs called at each timepoint in bulk and scRNAseq. a) Boxplot depicting the mean bulk RNAseq expression level in untreated cells for each DEG group from Figure 2a. d) Boxplot depicting the scRNAseq detection ratio in untreated cells for each DEG group from Figure 2a. e) Boxplot depicting the Log2 fold change across all timepoints of the bulkRNAseq for each DEG group from Figure 2a.

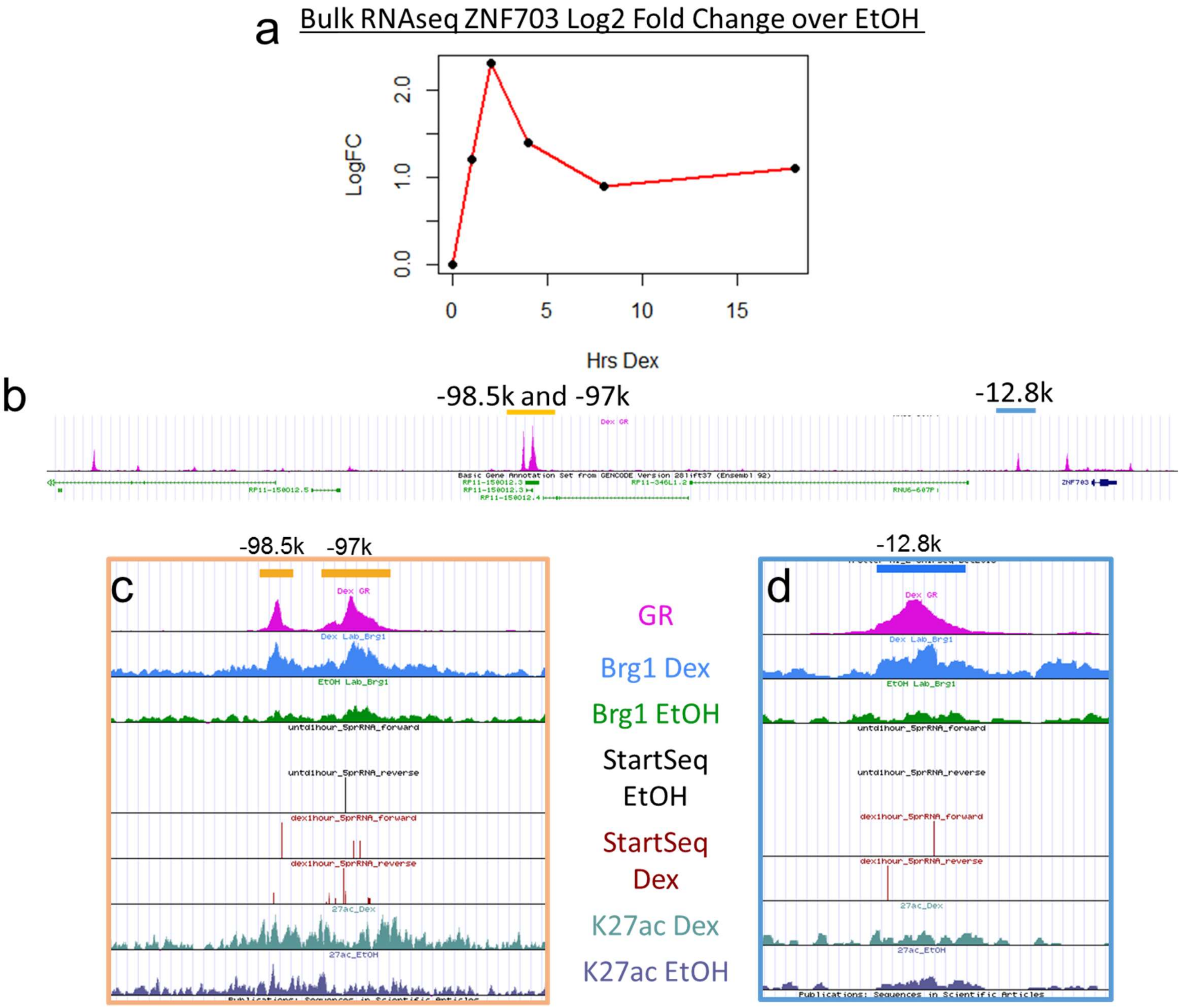

Supplemental Figure 3: a) Bulk RNAseq log2 fold change for ZNF703 across all Dex timepoints. b) Expanded browser shot depicting GR ChIP-seq coverage around the *ZNF703* gene locus. Several GR peaks are evident, major peaks at -98.5, -97, and -12.8 kb are highlighted. c-d) Zoomed in view of GR peaks -98.5kb/-97kb and -12.8kb upstream of the *ZNF703* TSS including BRG1 and H3K27ac ChIP-seq coverage +/- Dex and StartSeq +/- Dex.

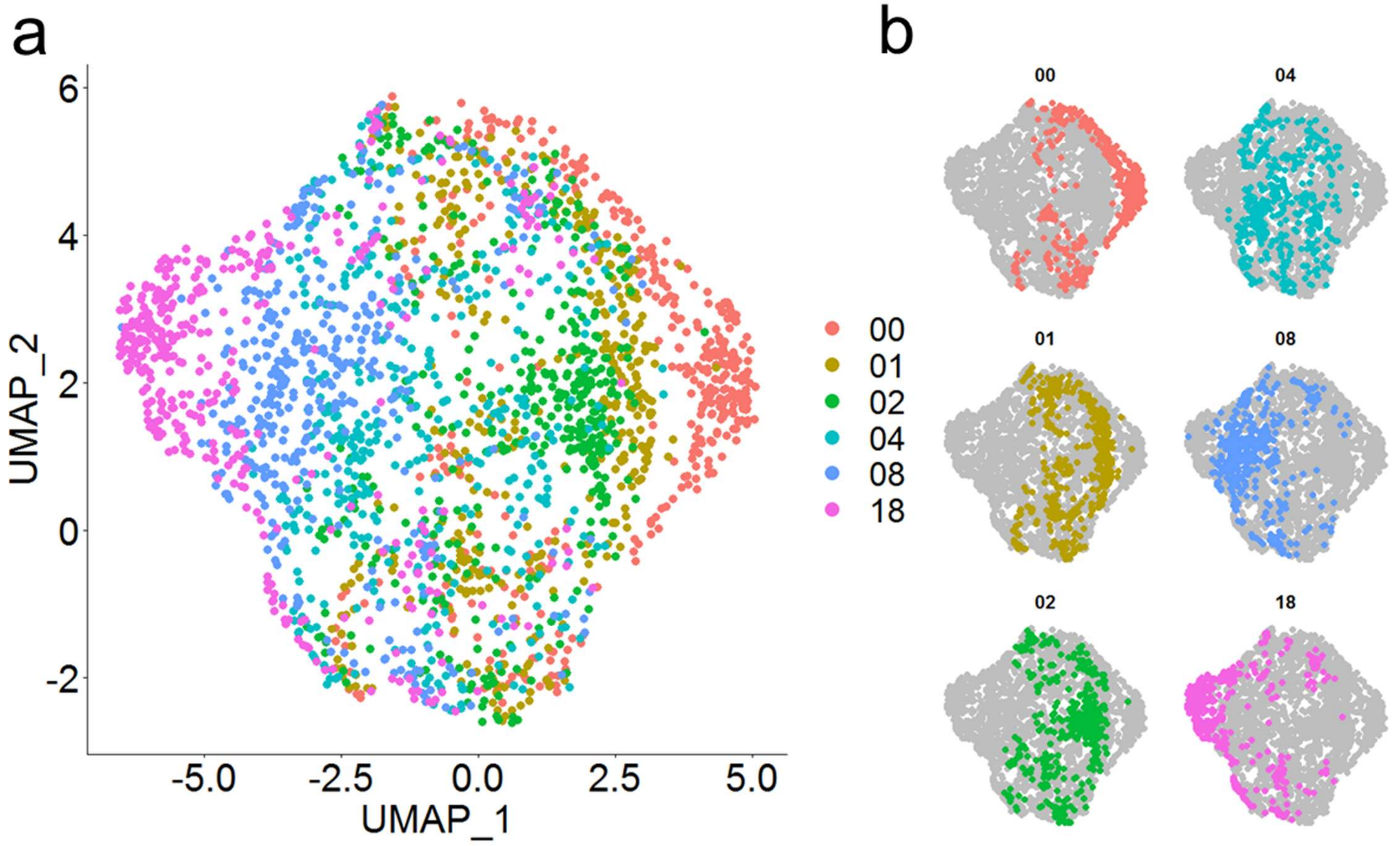

Supplemental Figure 4: a) UMAP plot of all 2400 cells from the scRNAseq color-coded by treatment timepoint. b) UMAP plot depicting each timepoint individually colored against all other cells.

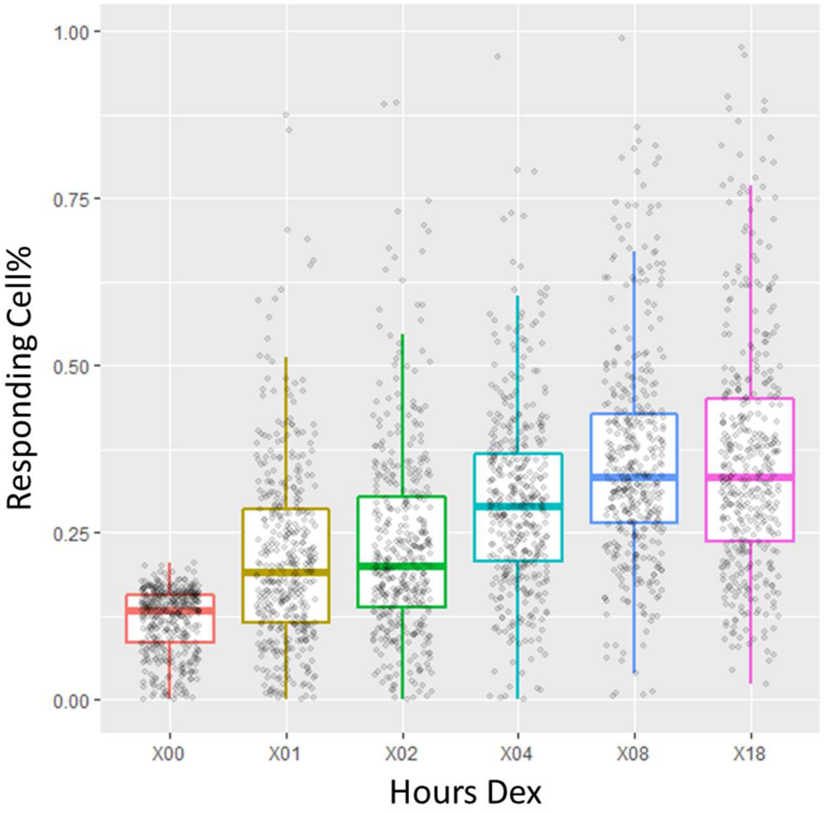

Supplemental Figure 5: Box and scatter pot depicting the Responding Cell% for each DEG. In this plot, each dot represents a DEG.

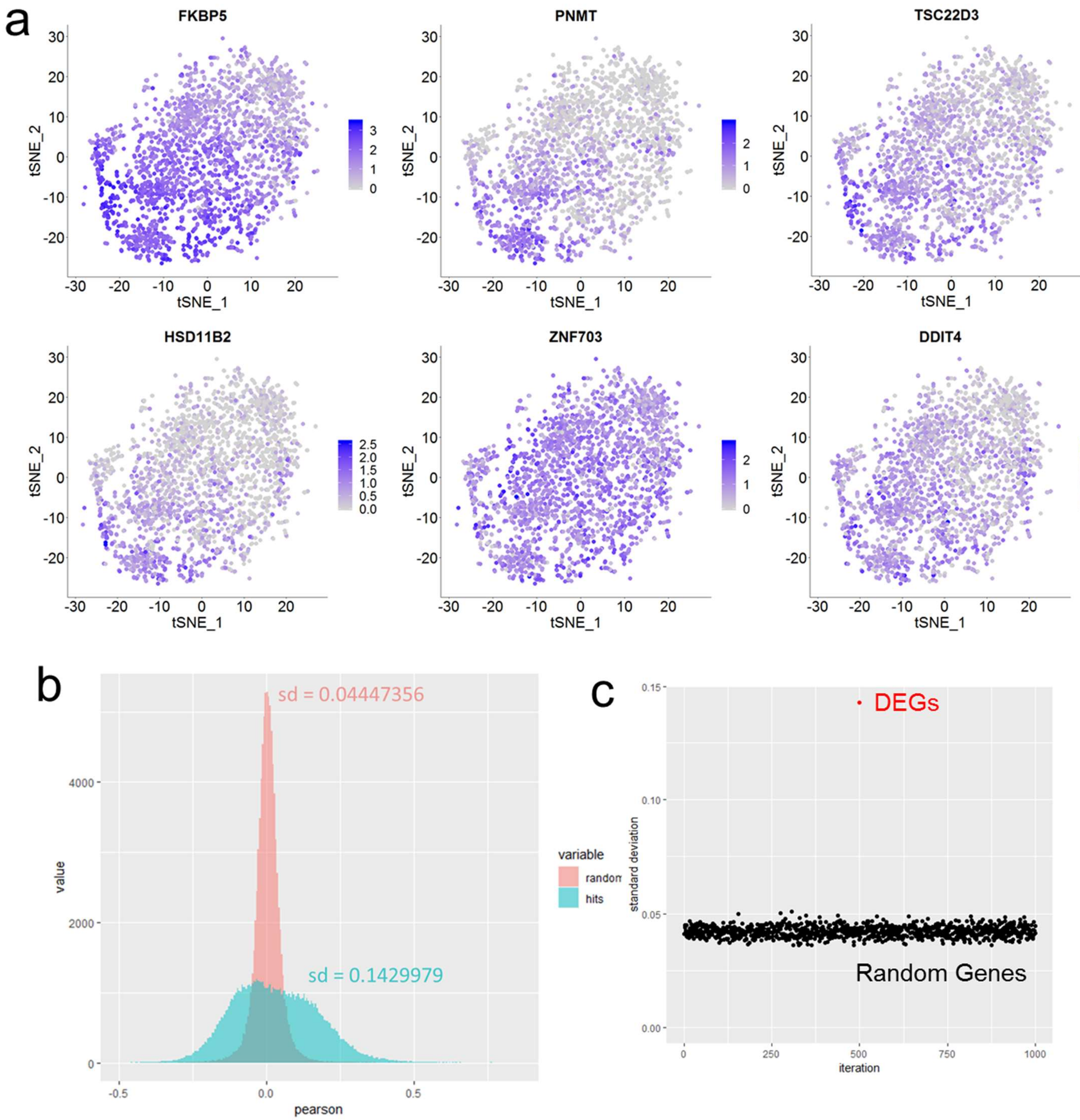

Supplemental Figure 6: a) scaled-log expression of DEGs modeled over tSNE plots. b) Histogram depicting all pairwise Pearson correlations of DEGs (cyan) compared to a random subset of genes expressed at similar levels (pink). c) Scatter plot depicting standard deviation of Pearson correlations of DEGs (red dot) compared to 1000 random sets of genes expressed at similar levels (black dots).

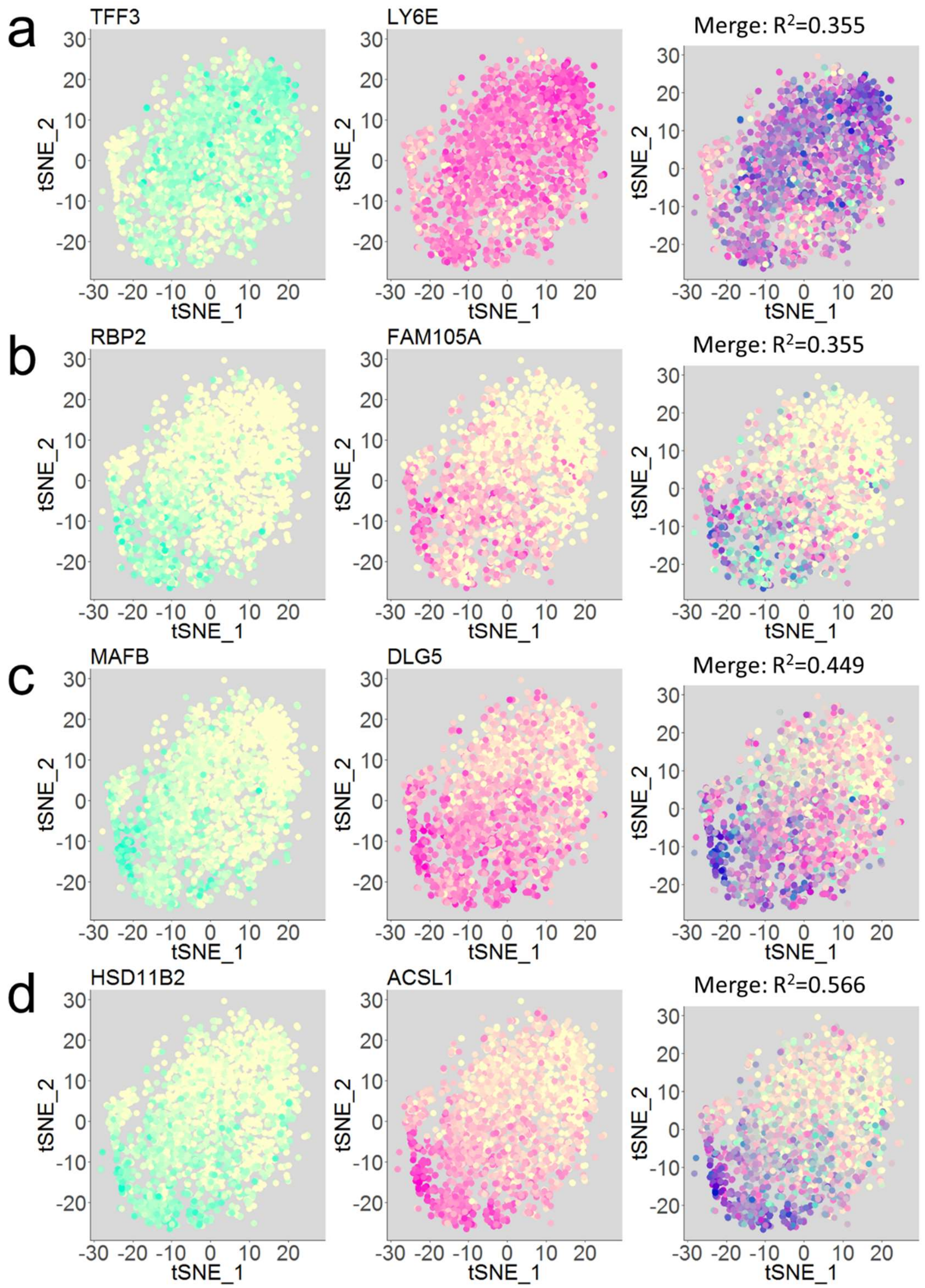

Supplemental Figure 7. a-d) tSNE plots designed to mimic co-localization in immunofluorescent images. In the merged image, dark blue indicates a high level of co-expression. R^2 value represents the Pearson correlation of the two genes. a) Hormone-repressed genes TFF3 (cyan) and LY6E (magenta) scaled-log expression modeled on a tSNE plots. b) Hormone-induced genes RBP2 (cyan) and FAM105A (magenta) scaled-log expression modeled on a tSNE plots. c) Hormone-induced genes MAFB (cyan) and DLG5 (magenta) scaled-log expression modeled on a tSNE plots. d) Hormone-induced genes HSD11B2 (cyan) and ACSL1 (magenta) scaled-log expression modeled on a tSNE plots.
