# Supplementary material for: Single-cell RNA sequencing reveals a heterogeneous response to Glucocorticoids in breast cancer cells
Source: Commun Biol. 2020 Mar 13;3:126. doi: 10.1038/s42003-020-0837-0 (PMC7070043; doi:10.1038/s42003-020-0837-0)
Supplement: Supplementary file 2 — Reporting Summary [file 42003_2020_837_MOESM2_ESM.pdf]

nature research

Corresponding author(s): Trevor K Archer

Last updated by author(s): 2020/2/7

Reporting Summary

Nature Research wishes to improve the reproducibility of the work that we publish. This form provides structure for consistency and transparency in reporting. For further information on Nature Research policies, see Authors & Referees and the Editorial Policy Checklist.

Statistics

For all statistical analyses, confirm that the following items are present in the figure legend, table legend, main text, or Methods section.

n/a	Confirmed	
☐	☒	The exact sample size (n) for each experimental group/condition, given as a discrete number and unit of measurement
☒	☐	A statement on whether measurements were taken from distinct samples or whether the same sample was measured repeatedly
☐	☒	The statistical test(s) used AND whether they are one- or two-sided<br>*Only common tests should be described solely by name; describe more complex techniques in the Methods section.*
☒	☐	A description of all covariates tested
☐	☒	A description of any assumptions or corrections, such as tests of normality and adjustment for multiple comparisons
☐	☒	A full description of the statistical parameters including central tendency (e.g. means) or other basic estimates (e.g. regression coefficient) AND variation (e.g. standard deviation) or associated estimates of uncertainty (e.g. confidence intervals)
☒	☐	For null hypothesis testing, the test statistic (e.g. F, t, r) with confidence intervals, effect sizes, degrees of freedom and P value noted<br>*Give P values as exact values whenever suitable.*
☒	☐	For Bayesian analysis, information on the choice of priors and Markov chain Monte Carlo settings
☒	☐	For hierarchical and complex designs, identification of the appropriate level for tests and full reporting of outcomes
☒	☐	Estimates of effect sizes (e.g. Cohen's d, Pearson's r), indicating how they were calculated

Our web collection on statistics for biologists contains articles on many of the points above.

Software and code

Policy information about availability of computer code

Data collection

No software was used for the collection of data

Data analysis

Software used for scRNAseq data analysis is described and referenced in our methods section, lines 454-471, excerpted below:

"Reads were parsed for cell barcode and UMI information, filtering out reads according to the following constraints: no 6 nucleotide barcode segment is more than a Hamming distance of 1 from a valid barcode block; all linker segments are within 1 nucleotide length of the expected value; the UMI has a length of 8 nucleotide; and only 1 mismatch is allowed within the ACG-GAC segments flanking the UMI. Reads with cell barcode information passing these filters were then aligned to the hg19 reference genome using STAR v2.5.2b31 and annotated with the Gencode comprehensive v28lift37. Uniquely-mapped reads were subsequently assigned to genes using featureCounts v1.5.332. PCR duplicates were removed using umi_tools33 in per-gene mode. The knee-calling algorithm from umi_tools was used to identify an appropriate total transcript count cut-off for cells in each sample, yielding a total of 4001 cells. Cells with mitochondrial gene percentage greater than 5% were removed, and cell counts were balanced between time points by randomly sub-setting each time point to 400 cells. Cyclone from the scran package34,35 was used to determine cell-cycle scores for all cells, and Seurat v336 was then use to normalize and scale the data such that the impacts of transcript counts, mitochondrial percent, and cell-cycle scores were regressed-out. Seurat and ggplot237 were used for data visualization."

Software used for bulk RNAseq analysis was described in our previous manuscript (https://elifesciences.org/articles/35073) which is referenced in the methods section of this study. The relevant excerpt:

"Adapter sequences were trimmed from RNA-seq reads using Cutadapt (Martin, 2011) and low quality reads were removed from analysis using Sickle (Joshi NA et al., 2011). Alignment was performed using STAR (Dobin et al., 2013) to generate coverage tracks and using Salmon (Patro et al., 2017) and to obtain gene counts for differential expression analysis using limma-voom (Law et al., 2014) with cutoffs of Fold Change > 1.5, p-value<0.01, and False Discovery Rate < 0.05."

For manuscripts utilizing custom algorithms or software that are central to the research but not yet described in published literature, software must be made available to editors/reviewers. We strongly encourage code deposition in a community repository (e.g. GitHub). See the Nature Research guidelines for submitting code & software for further information.

Data

Policy information about availability of data

All manuscripts must include a data availability statement. This statement should provide the following information, where applicable:

- Accession codes, unique identifiers, or web links for publicly available datasets
- A list of figures that have associated raw data
- A description of any restrictions on data availability

All bulk and single-cell RNAseq data have been submitted to GEO and will be made publicly available upon publication. Previously published NGS data that used in the manuscript is already publicly available at GEO. The following data availability statement is included in our manuscript:

"RNA-seq data generated for this publication have been deposited in NCBI's Gene Expression Omnibus (Edgar et al., 2002) and are accessible through GEO Series accession number GSE141834 (https://www.ncbi.nlm.nih.gov/geo/query/acc.cgi?acc=GSE141834). Our previously published data set is accessible through GSE112491 (https://www.ncbi.nlm.nih.gov/geo/query/acc.cgi?acc=GSE112491). "

Field-specific reporting

Please select the one below that is the best fit for your research. If you are not sure, read the appropriate sections before making your selection.

☒ Life sciences ☐ Behavioural & social sciences ☐ Ecological, evolutionary & environmental sciences

For a reference copy of the document with all sections, see nature.com/documents/nr-reporting-summary-flat.pdf

Life sciences study design

All studies must disclose on these points even when the disclosure is negative.

Sample size	For bulk RNAseq, we used 3 biological replicates for each timepoint. This is a widely used standard for bulk RNAseq experiments. For single-cell RNAseq, we isolated cells from individual cell preparations for each timepoint. We aimed to collect as many cells as possible for analysis, but decided to randomly down-sample each timepoint to 400 cells to balance the samples for easier statistical analysis and cleaner visualization of the data.
Data exclusions	Beyond down-sampling the number of single-cell RNAseq to 400 cells per timepoint, no data was excluded from the manuscript
Replication	As our experiment included a comparison of bulk and single-cell RNAseq, we used each data set to demonstrate the reproducibility of the other. Where the data sets differed, we attempted to validate and reproduce this findings using independent experiments, such as QPCR as described for ZNF703 in the manuscript.
Randomization	Our study focused on distinct hormone treatment timepoints. Samples were not randomly allocated into groups.
Blinding	Investigators were not blinded during data collection and analysis. Analysis was dependent on the logical organization of the data along the hormone treatment timecourse, and thus blinding and/or randomization would have prevented logical data analysis.

Reporting for specific materials, systems and methods

We require information from authors about some types of materials, experimental systems and methods used in many studies. Here, indicate whether each material, system or method listed is relevant to your study. If you are not sure if a list item applies to your research, read the appropriate section before selecting a response.

Materials & experimental systems

n/a	Involved in the study	
☒	☐	Antibodies
☐	☒	Eukaryotic cell lines
☒	☐	Palaeontology
☒	☐	Animals and other organisms
☒	☐	Human research participants
☒	☐	Clinical data

Methods

n/a	Involved in the study	
☒	☐	ChIP-seq
☒	☐	Flow cytometry
☒	☐	MRI-based neuroimaging

Eukaryotic cell lines

Policy information about cell lines

Cell line source(s)	We used human T47D A1-2 cells. A1-2 cells were obtained from Steven Nordeen and Dean Edwards, who originally generated the cell line (https://academic.oup.com/mend/article/3/8/1270/2713941).

Authentication	STR profiling was used to validate that the A1-2 cells genetically matched parental T47D cells as profiled by ATCC.
Mycoplasma contamination	A1-2 cells tested negative for mycoplasma contamination prior to use for the experiments described in the manuscript.
Commonly misidentified lines (See ICLAC register)	This study did not use any commonly misidentified cell lines.
